# A systematic review and meta-analysis on the ocular characteristics in children and adolescents with neurodevelopmental disorders

**DOI:** 10.1038/s41598-023-46206-9

**Published:** 2023-11-08

**Authors:** Sima Dastamooz, Clement C. Y. Tham, Jason C. S. Yam, Minghui Li, Stephen H. S. Wong, Cindy H. P. Sit

**Affiliations:** 1 Department of Sports Science and Physical Education, Faculty of Education, The Chinese University of Hong Kong, Hong Kong, China; 2 Department of Ophthalmology and Visual Sciences, Faculty of Medicine, The Chinese University of Hong Kong, Hong Kong, China

**Keywords:** Cognitive neuroscience, Development of the nervous system, Diseases of the nervous system, Visual system

## Abstract

To conduct a systematic review and meta-analysis of the association between children and adolescents with attention deficit hyperactivity disorder (ADHD) or autism spectrum disorder (ASD) and ocular characteristics. Systematic review with meta-analysis. Six databases (PubMed, Scopus, APA PsycInfo, Embase, EBSCOhost, and Cochrane library) were selected for a systematic literature search from database inception to July 2022. The observational studies assessing and reporting at least one outcome regarding ocular characteristics in children and adolescents with ADHD or ASD aged 6–17 were included. Studies in languages other than English, studies of adult or elderly human populations, and animal studies were excluded. The results were analyzed following the PRISMA guideline 2020. The findings of 15 studies, including 433 participants with ADHD, 253 participants with ASD, and 514 participants with typical development (TD), revealed that there were no significant differences in retinal nerve fiber layer, ganglion cell complex, and macular thickness between the ADHD group and the TD group. In subgroup analysis, significant differences in inferior ganglion cell (MD = − 3.19; 95% CI = [− 6.06, − 0.31], *p* = 0.03) and nasal macular thickness (MD = 5.88; 95% CI = [− 0.01, 11.76], *p* = 0.05) were detected between the ADHD group and the TD group. A significant difference in pupillary light reflex (PLR) was also observed between the ASD group and the TD group (MD = 29.7; 95% CI = [18.79, 40.63], *p* < 0.001). Existing evidence suggests a possible association between children and adolescents with ADHD or ASD and ocular characteristics. Given the limited number of studies, further research on a larger cohort is necessary to claim a possible diagnosis of ADHD or ASD through ocular characteristics.

## Introduction

Neurodevelopmental disorder (NDD) is a commonly-used term that refers to abnormal brain development, which causes impairments in communication, perception, and motor or behavioral skills. Autism spectrum disorder (ASD) and attention deficit hyperactivity disorder (ADHD) are the most common types of NDD^1^. Global statistics indicate that more than 7% of children have ADHD^2^, while less than 2% have ASD^3^. One-third of children with ASD also have ADHD symptoms^4^. They struggle with complications, which include motor dysfunction^5^, inattention^4^, lower cognitive ability, and slower reaction time^6^.

Evidence shows that the eye signals when there is a neurodegenerative disorder^6^. Retina parameters are used as structural indicators of axonal deformities and neurodegeneration diseases like Alzheimer's disease, multiple sclerosis, and Parkinson's disease^7,8^. Recently, researchers have suggested that retina features differ in patients with NDD^7,9,10^. In the 1990s, retinal ganglion cell complex (GCC) absence was explained as a mark of retinal engagement in cognitive function^7^. Autism neuropathology is related to a lack of neurogenesis and a deficit of neural resettlement^7^. A probable relationship exists between a lessening of thickness in the superior and nasal quadrants of the retinal nerve fiber layer (RNFL) and pathological neurodevelopmental issues among prematurely born infants^8^, while low birth weight is strongly associated with an increased risk of ASD^9,10^. Moreover, it is documented that systemic infection premature delivery, as environmental and biological factors, can cause ocular issues, for instance, strabismus and blurred vision^11,12^. The association between these environmental risk factors and the emergence of ADHD symptoms was ascertained in a recent umbrella review^13^. Modified brain development may simultaneously cause visual abnormalities and the advent of ADHD symptoms^14^. One reason would be the same embryonic origin of the brain and eye^6,15^.

A disease's presence is not only indicated by neural abnormalities but also by blood circulation parameters. Retinal microcirculation is known as a biomarker of cardiovascular disease^13^. Retinal microcirculation and cerebral tiny vessels are tightly associated as they have the same embryological origin and the same structural and functional features^8^. An examination of 3280 individuals using ophthalmic examination, Heidelberg Retina Tomography (HRT) imaging, and retinal vascular caliber quantities revealed that thinner retinal vessel caliber was related to a decrease in RNFL thickness^16^. This suggests that retina artery density might possibly be an indicator of central nervous system (CNS) abnormalities.

In the functional part of the eye, the locus coeruleus norepinephrine (LC-NE) system has been shown to be connected to pupillary response^15^. The LC, part of the sympathetic nervous system (SNS), plays a vital role in attention^17^, is the origin of norepinephrine in the brain, and is involved in arousal^18^. Brain function can be measured non-invasively by evaluating the pupil reflex, and this, in turn, can enable autism to be diagnosed early^15^.

Previous meta-analytic studies of anatomical abnormalities of the ocular structure are in conflict. Li et al. (2021) investigated the reduction of RNFL in ADHD based on four included studies. In contrast, Bellato et al. (2022) did not find any significant differences in RNFL thickness in patients with ADHD. They only ran an overall analysis on RNFL and did not conduct any subgroup analysis on the retinal segment; additionally, they included all age groups. The association of anatomical structure of the eye, specifically in children and adolescents with ADHD, is still unclear. Furthermore, none of the mentioned reviews considered the assessment of the alternation of the retinal circulatory system in patients with NDD. In functional ocular research, only one review covered pupillary light reflex (PLR) alterations in all age ranges in individuals with ASD^19^. This study attempted to fill the research gap detected in ocular literature in children and adolescents with NDD. Based on previous studies, we focused on the five most measured ocular characteristics in children and adolescents with NDD: retinal nerve fiber layer, macula, ganglion cell thickness, vascular abnormalities, and pupillary light reflex^12^. We compared these characteristics with those shown by their peers with typical development (TD). The empirical data were sorted in the qualitative section, and the quantitative difference was detected by conducting a meta-analysis.

## Methods

### Literature Search Strategy

The results were analyzed following the Preferred Reporting Items for Systematic Review and Meta-Analysis Protocols (PRISMA-P) Checklist. Six databases (PubMed, Scopus, APA PsycInfo, Embase, EBSCOhost, and Cochrane Library) were selected for a systematic literature search from database inception to April 2021 and an update search was conducted in July 2022. The advanced search covered all fields related to ADHD or ASD and ocular characteristics (“Autism*” OR “Attention Deficit Hyperactivity Disorder*” [All Fields] AND “Retinal nerve fiber layer” OR “Macula ganglion cell” OR “Vascular abnormalities” OR “Pupillary light reflex” OR “Ganglion cell complex” [All Fields]). The comprehensive details of the search strategy and search term used are shown Appendix 1. Moreover, the authors (SD and LMH) scanned the references of previous eligible studies to discover other studies associated with this topic.

### Study selection

Inclusion criteria were peer-reviewed English-language academic articles that reported cross-sectional or experimental studies of children and adolescents (aged 6–17 years) with ADHD or ASD. Studies in other languages, adult or elderly human populations, and animal studies were excluded.

**Figure 1:**
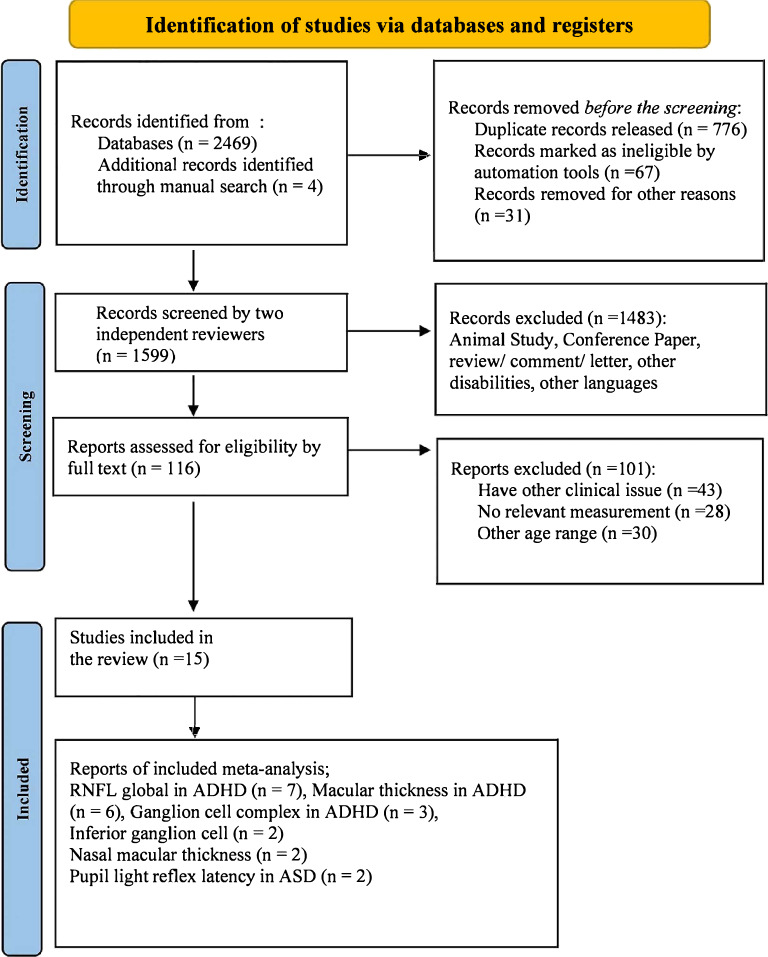
PRISMA flow study selection diagram, 2020.

To evaluate the accuracy of the search procedure, two reviewers (Dastamooz and Li) with expertise in studies of children and adolescents with NDD and ocular anatomy executed a multi-step search procedure and screened the titles, abstracts, and full-length texts. The reviewers independently conducted initial appraisals. We calculated the inter-rater reliability (K value) for reviewing abstracts and monitoring full texts used by the reviewers. Any discrepancy between the two reviewers was addressed by a third reviewer (Sit). There were 116 abstracts that met the inclusion criteria, and the inter-rater reliability was 0.87. Fourteen studies met the inclusion criteria, with an inter-rater reliability of 0.91 after full-text assessment. An additional manual search was conducted. Ultimately, 15 studies were selected for review and 11 for meta-analysis (see Fig. 1).

### Data extraction and quality assessment

A checklist was used for data extraction, which contained: (a) descriptive information, e.g., author information, date of publication, ethnicity; (b) study design; (c) participants' information such as age, sex, NDD types, diagnosis method, and sample size; (d) data collection characteristicsincluding Optical Coherence Tomography (OCT) equipment and OCT characteristics; and (e) significant findings.

### Quantitative analysis

Review Manager Software (V.5.4.1) was used to conduct the meta-analysis. The analysis model used for this meta-analysis was the random-effects model. The statistical analysis included: (1) mean (M), sample size (N), and standard deviation (SD), which were the primary approaches for estimating effect size (for analyzing RNFL, GCC, and macular thickness); (2) studies measuring one or more ocular characteristics domains were enrolled in the meta-analysis; (3) a holistic meta-analysis was conducted on RNFL, macula, and GCC, and then subgroup analyses were completed based upon 3mm to 6mm diameter ring the Early Diabetic Retinopathy Study (ETDRS) grid, which divided the retina into superior, nasal, inferior, and temporal or one to nine regions and clock-hour sectors (C) to evaluate RNFL, GCC, and macula; and (4) heterogeneity between studies was detected using the I^2^ test.

A value of I^2^ greater than 50% was considered heterogeneous; this was used to estimate the heterogeneity of the included studies. In the event of a significant result (*p* < 0.05), outliers were presumed based on 95% confidence intervals. The statistical significance level was set to *p* < 0.05. The results of the meta-analysis are illustrated as forest plots.

### Qualitative analysis

NVivo software (V 12) was used for qualitative analysis^20^. The Framework method was used to code the included studies to construct an inductive analysis in the discussion. First, the author read the discussion of the included studies to familiarize herself with the data. The data were then coded in NVivo to extract themes from each part. This developed the analytic framework and made identifying the relationship between themes easier. The framework was then applied to all the variables included in the systematic review and interpreted in the discussion.

### Quality assessment

The Newcastle–Ottawa Scale (NOS) for the quality of case–control studies in the meta-analysis was used to assess the methodological quality of the included studies^21^. The maximum score for the NOS scale is 9. In the present meta-analysis, the studies that scored ≥ 6 were considered comparatively high-quality studies. Dastamooz and Li independently assessed the methodological quality of the studies. Regarding discrepancies, the reviewers usually reached an accord through discussion. Otherwise, the third reviewer made the final decision (Table 1).

**Table 1:** The quality assessment for included studies.

Author, Year, and Country	Study design								Confounder	Total score out of 9	Overall investigation quality
	Is the case definition adequate?	Representatives of the cases	Selection of controls	Definition of control	Comparability of case and controls on the basis of design and analysis	Ascertainment of exposure	The same method of ascertainment for cases and controls	Non-response rate			
Aslan et al.^22^ (2020, Turkey)	Case–Control	1	1	0	1	1	1	1	0	6	High
Ayyildiz et al.^23^ (2019, Turkey)	Case–Control	1	0	0	1	1	1	1	0	5	Moderate
Bae et al.^24^ (2019, South Korea)	Case–Control	1	1	0	0	1	1	1	0	5	Moderate
Bodur et al.^25^ (2018, Turkey)	Case–Control	1	1	1	0	1	1	1	0	5	Moderate
Daluwatte et al.^26^ (2013, USA)	Case–Control	1	1	1	1	1	1	1	0	7	High
Fan et al.^27^ (2009, USA)	Case–Control	1	1	1	0	1	1	1	0	6	High
Garcia-Medina et al.^28^ (2017, Spain)	Case–Control	1	1	1	1	1	1	1	0	7	High
Garcia-Medina et al.^29^ (2020, Spain)	Case–Control	1	1	1	0	1	1	1	0	6	High
Grönlund et al.^30^ (2007, Sweden)	Case–Control	1	1	1	0	1	1	1	0	6	High
Hergüner et al.^15^ (2018, Turkey)	Case–Control	1	1	0	0	1	1	1	0	5	Moderate
Işık et al.^31^ (2020, Turkey)	Case–Control	1	1	0	0	1	1	1	0	5	Moderate
Lynch et al.^32^ (2018, USA)	Case–Control	1	1	0	1	1	1	1	0	6	High
Sánchez-Guillén et al.^29^ (2020, Spain)	Case–Control	1	1	0	1	2	1	1	0	7	High
Tarakcioglu et al.^33^ (2020, Turkey)	Case–Control	1	0	0	1	1	1	1	0	5	Moderate
Ulucan Atas et al.^34^ (2020, Turkey)	Case–Control	1	1	0	1	2	1	1	1	8	High

## Results

### Qualitative analysis

**Figure 2:**
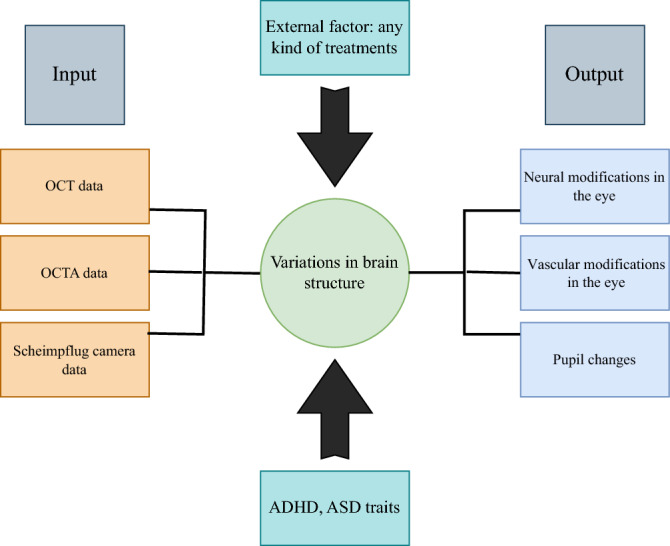
Framework scheme: these factors were extracted from the included studies for qualitative analysis.

This section analyzed the different ocular characteristics of groups and factors that affected them when they appeared in the discussion part of the studies. Framework analysis was used to summarize and interpret this data (see Fig. 2). This framework includes inputs, external factors, and participants' characteristics that could affect the brain and ocular features, which are evaluated in the discussion.

### Input (study characteristics)

The systematic review covered finalized articles that reported the findings of 15 studies conducted from database inceptions to July 2022. Each study was summarized and described in detail in Table 2. Geographically, eight studies were conducted in Asia, three in Europe, and three in the USA. In this review, all the studies utilized a case–control design.

**Table 2:** Descriptive characteristics of included studies.

Author and year	Design	Diagnostic methods	Participants characteristics		Device	Refraction error SE (diopter)	Parameters	Main findings
			Sample size, and Gender	Mean age				
Aslan et al.^22^ (2020, Turkey)	Case–Control	K-SADS-PL-T: DSM-IV	ADHD: 32 (M-72%) TD: 43 (M-72%)	ADHD:9.19 ± 1.93 TD: 9.35 ± 1.65	Cirrus OCT: Sirius 3D rotating Scheimpflug camera topography	NA	Pupillary light reflex velocity RNFL thickness PLRV and RNFL Correlation	PLRV and RNFL are Correlated (*P* = 0.003)
Ayyildiz et al.^23^ (2019, Turkey)	Case–Control	K-SADS-PL	ADHD: 30 (M-63%) TD: 30 (M-50%)	ADHD:142.89 ± 24.31 (Month) TD:153.13 ± 24.73 (Month)	Spectralis OCT	NA	RNFL thickness Macula thickness Anterior segment structure	Increase in axial length (*P* = 0.04) Decrease in Corneal curvature Radius (*P* = 0.03) Increase in Central corneal thickness (*P* = 0.005)
Bae et al.^24^ (2019, South Korea)	Case–Control	K-ARS	ADHD: 12 (M-33%) TD: 13 (M-15.38%)	ADHD:9.8 ± 2.1 TD: 10.6 ± 1.9	Cirrus OCT: Philips MRI scanner	ADHD: − 1.5 ± 2.1 (right) − 1.2 ± 2.2 (left) TD: − 1.9 ± 1.7 (right) − 2.0 ± 1.6 (left)	Macular thickness	Increase in macular thickness inner ETDRS ring (*P* = 0.04) and Outer ETDRS ring (*P* = 0.05, right; and *P* = 0.02, left)
Bodur et al.^25^ (2018, Turkey)	Case–Control	K-SADS-PL-T: DSM-IV	ADHD: 16 (M-81%) TD: 31 (M-15.38%)	ADHD:111.62 ± 27.05 (Month) TD: 127.19 ± 24.39 (Month)	iVUe 100	NA	RNFL GCL ONT	Decrease in ganglion cell layer (*P* = 0.0001, right eye; *P* = 0.002, left) Decrease in optic nerve thicknesses (*P* = 0.015, left)
Daluwatte et al.^26^ (2013, USA)	Case–Control	ADOS: DSM-IV	ASD: 152 (M-89%) NDD: 36 (M-75%) TD: 107 (M-74%)	ASD: 10.7 ± 3.4 NDD: 9.9 ± 3.0 TD: 10.9 ± 2.9	The binocular Pupillography recording system	NA	Resting pupil diameter, PLR latency, constriction, Constriction time, Redialation time, constriction velocity, Redialation velocity, heart rate	Increase in PLR latency (*P* < 0.0001) Decrease in constriction time (*P* = 0.0007) Decrease in redialation time (*P* = 0.01)
Fan et al.^27^ (2009, USA)	Case–Control	PDD-NOS	ASD: 24 (M-92%) TD: 44 (M-48%)	ASD: 12.9 ± 4.3 TD: 10.4 ± 2.7	A binocular pupillogram recording system	NA	Pupillary light reflex latency PLR constriction Anisocoria Contraction anisocoria	Decrease in pupillary light reflex latency (*P* = 0.013)
Garcia-Medina et al.^28^ (2017, Spain)	Case–Control	DSM-V	ASD: 27, TD:31 ASD: 27, TD:62	ASD: 13.70 ± 3.03 TD: 13.70 ± 3.03	Spectralis OCT TruTrack Active Eye Tracking	< 6 spherical diopters and < 2.5 cylinder diopters	Total retina	In pRNFL measurement increase in temporal inferior (*P* = 0.03), nasal inferior (*P* = 0.01), inferior (*P* = 0.01). In macular measurement increase in total retina (P = 0.04), total inner retina (*P* = 0.04)
Garcia-Medina et al.^29^ (2020, Spain )	Case–Control	DSM-IV	ASD: 13 (M-77%) TD:14 (M-71%)	ASD: 16.615 ± 2.987 TD: 16.857 ± 4.055	Cirrus 5000 device with Angioplex	NA	Full retina thickness Peripapillary retinal nerve fiber layer Vessel density Capillary perfusion density	Decrease in peripapillary perfusion density (*P* = 0.029) Increase in FLUX index (*P* = 0.037)
Grönlund et al.^30^ (2007, Sweden)	Case–Control	DSM-IV	ADHD + stimulants:42 (M-88%) TD: 50 (M-88%)	ADHD:12 TD: 11.9	Orbit, IOTA Inc Sundsvall eye-tracking technology KM- Bok's chart Monolateral cover test TNO random-dot test RAF ruler, Near point of Accommodation Auto refractometer	Myopia ≥ 0.5D or hyperopia ≥ 2.0 D	Visual acuity, strabismus, and ocular motility Near the point of convergence Refraction under cycloplegia, ocular dimensions	Decrease in visual acuity (*P* = 0.032) Decrease in heterophoria (*P* = 0.038) Decrease in subnormal stereovision (*P* = 0.016) Decrease in abnormal convergence (*P* = 0.031) Decrease in astigmatism (*P* = 0.03) Decrease in visuoperceptual problems (*P* = 0.007) Decrease in optic discs and neuroretinal rim areas (*P* < 0.0001) Decrease in tortuosity of retinal arteries (*P* = 0.0002)
Hergüner et al.^15^ (2018, Turkey)	Case–Control	DSM-IV	ADHD: 45 (M-84%) TD: 45 (M-84%)	ADHD: 8.6 ± 1.9 TD: 8.9 ± 2.1	Spectralis OCT	ADHD: − 0.17 ± 0.5 TD: − 0.24 ± − 0.6	RNFL thickness Macular thickness Correlation with CPRS-R: S SDQ:P	Decrease in nasal RNFL (*P* = 0.027) Inattention is correlated with Temporal inferior thickness (*P* = 0.002)
Işık et al.^31^ (2020, turkey)	Case–Control	K-SADS-PL-T DSM-IV	ADHD: 58 (M-71%) TD:44 (M-73%) ADHD + MPH:45 (M-47%)	ADHD: 9 ± 2.41 ADHD + MPH: 9.02 ± 2.19 TD: 10.85 ± 2.21	Cirrus OCT	NA	Intraocular pressure Macular thickness Retinal nerve fiber layer Ganglion cell layer thicknesses Intraocular pressure	Increase in Left IOP (*P* = 0.011)
Lynch et al.^32^ (2018, USA)	Case–Control	DSM-IV	ASD: 10 (M-80%) TD: 12 (M-58%)	ASD: 16.15 TD: 15.20	FaceLAB5VC eye-tracking technology	NA	Baseline pupil diameter latency to maximum constriction Pupil diameter at the maximum constriction, latency of return to baseline pupil diameter	Increase in Pupillary light reflex maximal latency (*P* = 0.04) Increase in latency of return to baseline pupil diameter (*P* = 0.02)
Sánchez-Guillén et al.^29^ (2020, Spain)	Case–Control	DSM	ADHD:9 (M-83%) TD: 23 (M-70%) ADHD + MPH:14 (M-83%)	ADHD: 11.9 ± 3.3 ADHD + MPH:12.4 ± 3 TD: 11.4 ± 3.1	Cirrus 5000 device with Angioplex	NA	Macular thickness Retina Ganglion cell complex, Retinal nerve fiber layer	Decrease in macular central thickness (*P* = 0.013)
Tarakcioglu et al.^33^ (2020, Turkey)	Case–Control	NA	ADHD + MPH: 53 (M-77%) ADHD: 40 (M-80%)	ADHD + MPH: 10.7 ± 1.9 ADHD: 9 ± 1.5	OCT RT XR Avanti with Angio Vue software	ADHD + MPH: ± 3 ADHD: ± 3	Foveal avascular zone Vessel density	Increase in flow area (choriocapillaris) at ADHD + MPH (*P* = 0.03) Increase in superficial parafoveal thickness ADHD + MPH(*P* = 0.01) Increase in deep parafoveal thickness ADHD + MPH (*P* = 0.01)
Ulucan Atas et al.^34^ (2020, Turkey)	Case–Control	K-SADS-PL-T/DSM-IV	ADHD: 37 (M-84%) TD: 37 (M-62%)	ADHD: 9.5 ± 2.2 TD: 9.4 ± 1.6	Nidek RS-3000 OCT device	ADHD ≤ ± 0.5 TD ≤ ± 0.5	Contrast sensitivity levels RNFL thickness Ganglion cell complex	Decrease in nasal RNFL (*P* = 0.006) Decrease in contrast sensitivity (*P* < 0.001)

*RNFL* Retinal nerve fiber layer thickness, *PLRV* Pupillary light reflex velocity, *GCL* Ganglion cell layer, *ONT* optic nerve thicknesses, *CCT* Central corneal thickness, *AL* Axial length, *CR* Corneal curvature radius, *CPRS-R:S* Conners’ Parent Rating Scale-Revised: Short, *SDQ:P* Strengths and Difficulties Questionnaire: Parent Form.

Various OCT devices captured the ocular characteristics used as input for these studies. These included Cirrus, Spectralis, iVUe 100, RT XR Avanti, and Nidek RS-3000, which were supported and analyzed by Angio Vue software; and the OCTA device, like the Cirrus 5000 device with Angioplex software, 3D rotating Scheimpflug camera topography, and the Binocular Pupillography recording system.

### ASD and ADHD traits

The sample involved 1,200 children and adolescents aged 6 to 17 years (ADHD = 433, ASD = 253, TD = 514). Of the 15 studies we considered, five were conducted on groups with ASD and 10 on groups diagnosed with ADHD, according to the criteria used in the Diagnostic and Statistical Manual of Mental Disorders fifth edition (DSM-V)^35^. The Schedule of Affective Disorders and Schizophrenia for School-Age Children-Present and Lifetime Version (K-SADS-PL) was also used to identify individuals with ADHD^19^, and the Autism Diagnostic Observation Schedule (ADOS) was used to diagnose ASD. The Conner's Parent Rating Scale-Revised (CPRS-R: S) and SDQ: P, teacher, and parent declaration forms were used to evaluate the severity of ADHD symptoms^15^.

### Brain structure

The Philips MRI scanner, 3 Tesla (Philips; Eindhoven, the Netherlands), evaluated the cortical thickness in the ADHD versus the TD group. This study was the only study that assessed the association between brain structure and macular changes in children and adolescents with ADHD^36^.

### Output

The studies investigated the RNFL, GCC, macular thickness, PLR, and retina artery vascularization density. In ASDs, three studies evaluated the PLR^21,24,37^, and only one evaluated the retinal condition^29^. In ADHDs, seven studies measured RNFL thickness, six assessed macular thickness, three evaluated GCC, and two calculated the optic disc and intraocular pressure. Several variables (corneal thickness, axial length, contrast sensitivity, color perception, visual acuity, pupil velocity, fovea thickness, the association between pupillary velocity and RNFL thickness, and the association between macular thickness and cortical thickness, and foveal avascular zone area and perimeter) were estimated in only one study each^33^. The study participants were excluded if the refraction error exceeded ± 3 diopters^33^.

### External factor

Information processing and neurotransmission can be affected by external factors such as medication. In four studies^36,38,39^, in the ADHD group, medication was considered an element that would affect the ocular variations. In the other studies, medication was included in the criteria for exclusion. In most of these studies, participants were treated with methylphenidate (MPH)^36,38,39^. As a psychostimulant, this drug enhanced the control of the hyperactive symptoms by boosting the inhibitory potential of the prefrontal cortex^33^. A study found that choriocapillaris, superficial parafoveal thickness, and deep parafoveal thickness in children with ADHD treated with MPH were thicker than in a group with ADHD who did not receive any external treatment^33^.

### Meta-analysis for comparison of the retinal changes in ADHD and ASD

**Figure 3:**
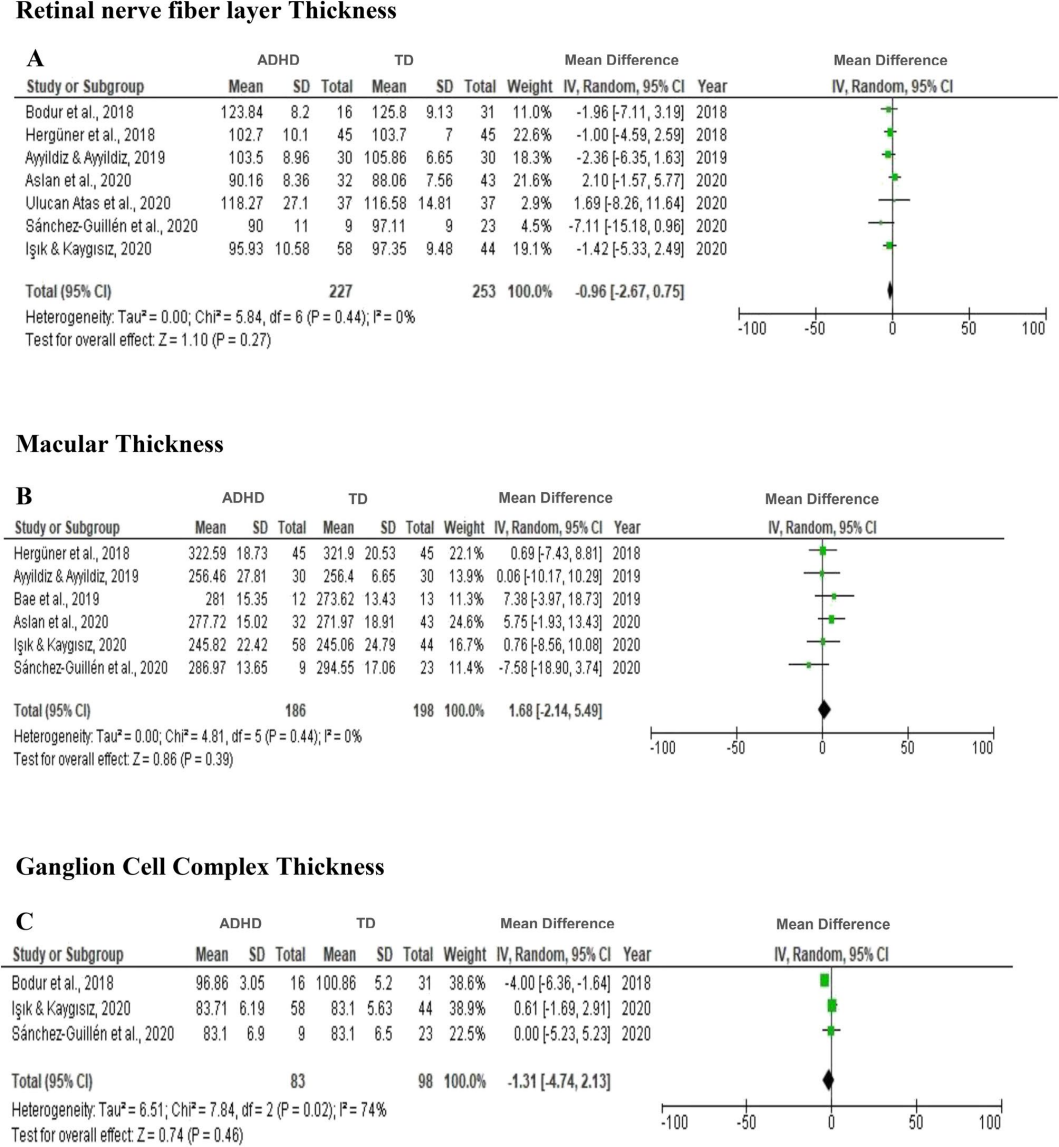

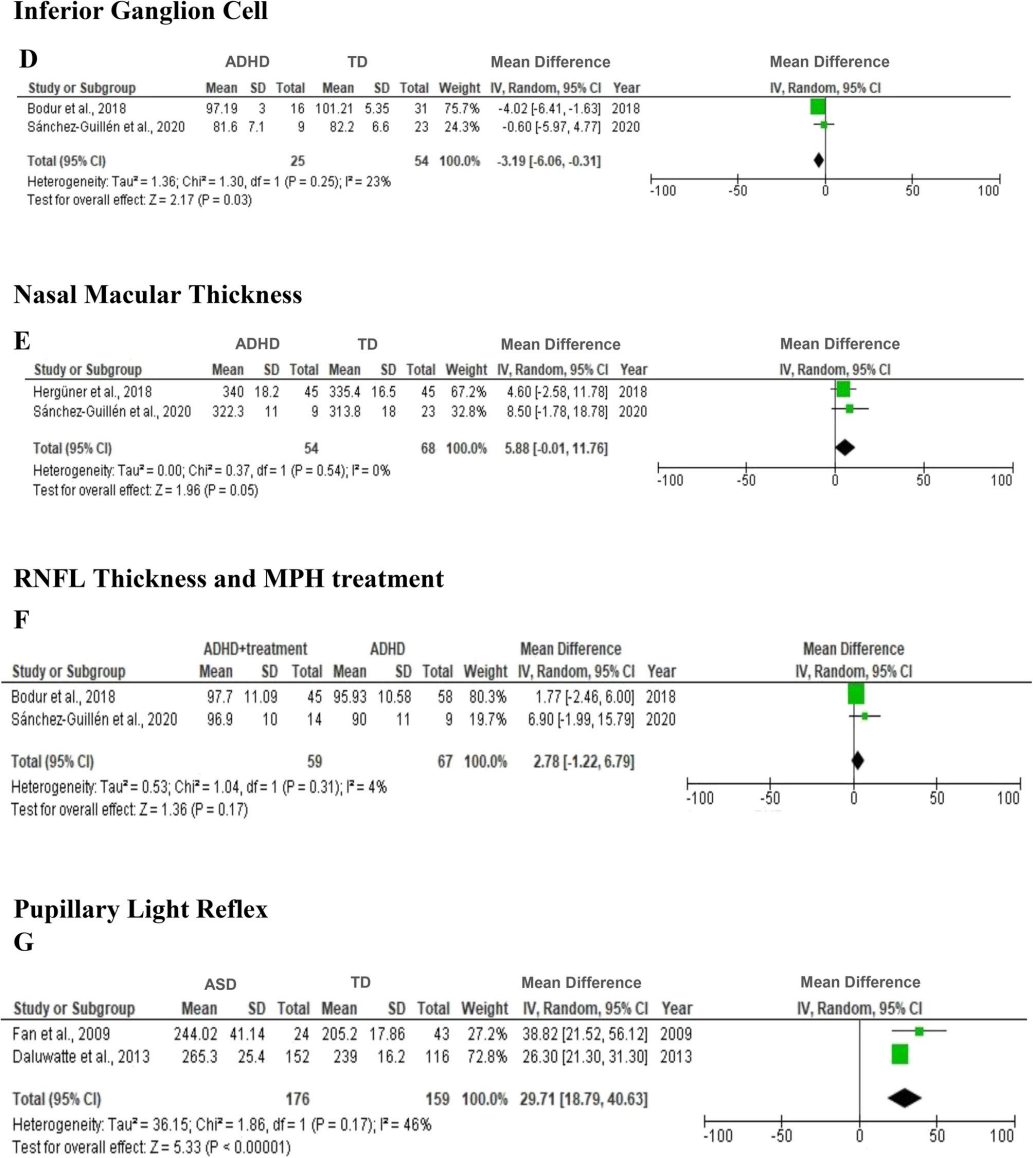
Forest plot for meta-analysis regarding the comparison between ADHD, and TD (**A**–**E**), ADHD with treatment (**F**), and ASD and TD (**G**).

Of the 15 studies covered in this review, 11 case–control studies were judged suitable for meta-analysis. The meta-analysis included 433 patients with ADHD, 253 patients with ASD and 514 TD. In the meta-analysis, data from children with ADHD, the RNFL thickness (7 studies), the GCC thickness (3 studies), and the macular thickness (6 studies) were included. In subgroup meta-analysis, inferior ganglion cell and nasal macular thickness, two studies in patients with ADHD were evaluated. The PLR of two studies was the only variable in the current meta-analysis, which considered children and adolescents with ASD. Figure 3 shows the meta-analysis results, indicating the effect size of ADHD and ASD on ocular characteristics.

In Fig. 3A–C, between-group analyses did not identify any significant differences in average RNFL (MD = − 0.96; 95% CI = [-2.67, 0.75], *p* = 0.27) (A), macular thickness (MD = 1.68; 95% CI = [− 2.14, 5.49], *p* = 0.39) (B), or GCC (MD = − 1.31; 95% CI = [− 4.74, 2.13], *p* = 0.46) (C).

Subgroup meta-analysis indicated that the ADHD group had significantly thinner inferior GCC thickness than the TD group (MD = − 3.19; 95% CI = [− 6.06, − 0.31], *p* = 0.03), with indications of non-significant heterogeneity (I^2^ = 23%, *p* = 0.05). The ADHD group also had significantly greater nasal macular thickness than the TD group (MD = 5.88; 95% CI = [− 0.01, 11.76], *p* = 0.05), with indications of non-significant heterogeneity (I^2^ = 0%, *p* = 0.54) (Fig. 3D,E). Methylphenidate, as a pharmacological treatment, appeared to thicken the RNFL. However, this modification was not significantly different between the ADHD group with treatment and without treatment (MD = 2.78; 95% CI = [− 1.22, 6.79], *p* = 0.17) (Fig. 3F,G). The ASD group also had significantly longer PLR than the TD group (MD = 29.7; 95% CI = [18.79, 40.63], *p* < 0.00001) with heterogeneity (I^2^ = 46%, *p* = 0.17).

### Publication bias

Chapter Five of the Cochrane Library Handbook recommends that the minimum number of studies included in the tests for funnel plot asymmetry or more advanced regression-based assessments should be at least ten, as the use of a smaller number of studies in a meta-analysis would decrease the power of the test to differentiate a fundamental asymmetry^40^. Therefore, assessing publication bias in this systematic review and meta-analysis was not currently feasible. As an alternative, we used NOS to assess the methodological quality of the included studies. Nine studies (60% of included studies) were rated as high quality, and six studies (40% of included studies) were rated as moderate quality (Table 1).

## Discussion

Our current review was the first comprehensive study to investigate the ocular characteristics of children and adolescents with ADHD or ASD, based on 15 studies previously conducted in this area. Our meta-analysis revealed no significant differences in global RNFL, GCC, and macular thickness between the ADHD group and the TD group. However, the ADHD group had significantly thinner inferior GCC and thicker nasal macular thickness. In children and adolescents with ASD, the PLR was significantly longer than in the TD group. Our results differed from those in Li's review (2021), which found a significant reduction in global retina thickness in children with ADHD, and in line with Bellato's review (2022), which failed to find a significant difference in global RNFL thickness between ADHD and TD group.

A cohort study of 2235 participants with TD detected an association between thinner RNFL and GCC with lower gray matter density in the primary visual cortex. GCC thickness was also strongly associated with cognitive function. Specifically, the GCC was associated with the gray matter density of the thalamus, close to the lateral geniculate nucleus in the middle of the visual pathway^41^. Referring to the direction of variations that occurred in the brain and eye, it was found that, in the retina layer, apoptosis in the GCC may lead to anterograde deterioration of the visual pathway, causing thinner RNFL and finally leading to atrophy of the visual cortex in the occipital lobe^42^. Conversely, injury in the visual cortex may result in variation of the optic nerve and retinal layer^42^. These results echoed our findings in the meta-analysis that inferior GCCs of children and adolescents with ADHD significantly decreased when compared with the TD group.

In contrast, nasal macular thickness in the ADHD group was significantly thicker than in the TD group. In embryological terms, retinal cells originate from the neuroepithelium and surface ectoderm and have a common origin with the brain. The RNFL is constructed from unmyelinated axons and is considered the equivalent of cerebral gray matter. Neuroimaging analyses have identified cerebral gray matter depletion in children with ADHD, explaining neural reductions in the eye^43^. However, the bilateral increase in macular thickness in ADHD was significantly associated with the thickness of regions in the parietal and frontal lobes^36^. One study found that the frontal cortex in children with ADHD was thicker than the parietal lobe^24^. Dysfunction in brain pruning in developing human ages is related to the thickness of the prefrontal cortex in a developing brain^37^, and maturation in human cognitive abilities is associated with pruning^36^. Controlling, organizing, and associating relevant information are critical roles in the frontal cortex^36^, while the primary function of the parietal cortex is to receive information from sensory stimuli from the entire body^44^. Deficiency in the frontoparietal circuit is associated with attention deficits^38,39^.

Adolescents with ASD often have a higher-than-normal blood vessel density in the eye. This may be caused by neuroinflammation^41^, abnormal parenchyma overgrowth^45^, or unusual vascular changes (such as blood–brain barrier disorder, e.g., blood-retina barrier, and angiogenesis^46,47^. By contrast, in adolescents with ASD, the Flux index is less intense, and the Flux index is positively correlated with RNFL thickness. Because of parenchymal metabolic demands, a biological healthy retina tissue is responsible for autoregulation of the blood FLUX, and any malfunction may be related to ASD physiology^48^.

Our review found that the PLR latencies were more prolonged in children and adolescents with ASD. Optic nerve atrophy^49^ and demyelination^50^ are associated with PLR latencies. ASD is not a primarily demyelinating disease, but MRI images have shown abnormal white matter signals at subcortical and posterior hyperintensities situated in the temporal poles^51^. An alternative explanation is the critical role of the cerebellum in modulating sensory input^52^. A reduction in the density of the Purkinje cells located in the cerebellum has been demonstrated in postmortem neuroanatomical investigations of individuals with ASD^53^. These findings have also been supported by diffusion tensor imaging^54^ and fMRI studies^55^. The involvement of the cerebellum in PLR has also been confirmed in animal studies^56^.

Medication, as an external factor, was considered certain patient specifications in some studies. The hyperactive dopamine (DA)/norepinephrine (NE) hypothesis could be an appropriate explanation for ADHD etiopathology^57^. Meanwhile, for individuals with ADHD, treatment with amphetamine and methylphenidate drugs as a psychostimulant has been suggested. These treatments block DA and NE re-uptake by inhibiting the transporters of these neurotransmitters (NTs). Stimulating alpha-adrenergic receptors causes induction of mydriasis through DA and NE^58^. High-dose MFD has a degenerative impact on corneal and retinal tissues by stimulating the dopaminergic pathway and inducing morphological variations^59^.

The present systematic review and meta-analysis found that, compared with the TD group, the ADHD group had thinner inferior GCC thickness and thicker nasal macular thickness. The ASD group also experienced longer PLR than the TD group. The strengths of our review are that it provides a unique interpretation of ocular abnormalities between children and adolescents with ADHD and ASD, and TD using a meta-analysis approach and that it is the first review that comprehensively covers the nature of ocular characteristics in two major NDD types.

The review has limitations, including the lack of randomized control trial studies, clinically referred population sampling (Berkson's bias), and limited evidence of retinal characteristics among children and adolescents with ASD. In addition, some of the studies included in this review and meta-analysis did not provide detailed information about the RNFL protocol, so the scan procedure information was not provided. Given the limited number of studies currently addressing this topic, our significant findings were based on a small dataset and should be further validated in future research. Nevertheless, the thought has improved our understanding of anatomical and functional ocular constructions in children and adolescents with ADHD and ASD. It may help to hasten the design and implementation of non-pharmaceutical interventions aimed at improving the symptoms exhibited by these groups.

In conclusion, we found significant differences in inferior GCC and nasal macular thickness in children and adolescents with ADHD when compared with the TD peers. Further research on a larger cohort is recommended to claim possible diagnoses of ADHD or ASD through ocular characteristics.

### Supplementary Information

Supplementary Information.

## Data Availability

Data are available by request from the corresponding author.
