# Supplementary material for: A systematic review and meta-analysis on the ocular characteristics in children and adolescents with neurodevelopmental disorders
Source: Sci Rep. 2023 Nov 8;13:19397. doi: 10.1038/s41598-023-46206-9 (PMC10632382; doi:10.1038/s41598-023-46206-9)
Supplement: Supplementary file 1 — Supplementary Information. [file 41598_2023_46206_MOESM1_ESM.docx]

("retina*" OR "retinal nerve fiber layer" OR "[fovea](https://www.ncbi.nlm.nih.gov/mesh/68005584)" OR "[retinal bipolar cells](https://www.ncbi.nlm.nih.gov/mesh/68051245)" OR "[ganglion cells](https://www.ncbi.nlm.nih.gov/mesh/68012165) complex" OR "[retinal horizontal cells](https://www.ncbi.nlm.nih.gov/mesh/68051248)" OR "[retinal cone photoreceptor cells](https://www.ncbi.nlm.nih.gov/mesh/68017949)" OR "[retinal rod photoreceptor cells](https://www.ncbi.nlm.nih.gov/mesh/68017948)" OR "retinal vessels" OR "static retinal vessel" OR "static retinal vessel analysis" OR "retinal blood vessels" OR "retinal arteriolar narrowing" OR "retinal arteriolar narrowing" OR "retinal arteriolar caliber" OR "retinal venular caliber" OR "retinal vasculature" OR "retinal vascular caliber" OR "retina/arteriovenous ratio" OR "retinal microcirculation" OR "retinal vessel diameter*" OR "dynamic retinal vessel analysis" OR "retinal arteriolar dilatation" OR "retinal venular dilatation" OR "retinal flicker-light response" OR "choroid" OR "macula volume" OR "macula thickness") AND ("ADHD" OR "attention deficits/ hyperactivity" OR "Autism" OR "Autistic Disorder" OR "ASD" OR "Autism Spectrum Disorders" OR "high function autism" OR "Asperger Disorder")
